# Discovery of *SOX5* as a New Causative Gene for Atrial Fibrillation

**DOI:** 10.3390/diagnostics16010059

**Published:** 2025-12-24

**Authors:** Dao-Liang Zhang, Xing-Biao Qiu, Ning Li, Yuan-Yuan Ding, Chen-Xi Yang, Zun-Ping Ke, Ying-Jia Xu, Yi-Qing Yang

**Affiliations:** 1 Cardiac Arrhythmia Center, Fuwai Hospital, Chinese Academy of Medical Sciences, Shenzhen 518057, China; 2 Department of Cardiology, Shanghai Chest Hospital, Shanghai Jiao Tong University School of Medicine, Shanghai 200030, China; qiuxingbiao@hotmail.com; 3 Department of Cardiology, Putuo Hospital, Shanghai University of Traditional Chinese Medicine, Shanghai 200062, China; frankleelx@163.com; 4 Shanghai Health Development Research Center, Shanghai Medical Information Center, Shanghai 200031, China; dingyuanyuan@shdrc.org; 5 Department of Cardiology, Shanghai Fifth People’s Hospital, Fudan University, Shanghai 200240, China; cxyang21@m.fudan.edu.cn (C.-X.Y.); xuyingjia@5thhospital.com (Y.-J.X.); 6 Department of Geriatrics, Shanghai Fifth People’s Hospital, Fudan University, Shanghai 200240, China; kzprenmin@163.com; 7 Department of Cardiovascular Research Laboratory, Shanghai Fifth People’s Hospital, Fudan University, Shanghai 200240, China; 8 Department of Central Laboratory, Shanghai Fifth People’s Hospital, Fudan University, Shanghai 200240, China

**Keywords:** arrhythmia, atrial fibrillation, molecular genetics, SOX5, transcriptional regulation

## Abstract

**Background/Objectives:** Atrial fibrillation (AF), characteristic of chaotic atrial electrical activity along with ineffective atrial systole, remains the most frequent sustained cardiac dysrhythmia, with an overall lifetime risk for AF being approximately 15% to 40% in the global population. AF is associated with substantially enhanced risks for multiple adverse clinical outcomes, including thromboembolic cerebral stroke, dementia, chronic kidney disease, myocardial infarction, cardiac failure, and even premature cardiac demise. Although remarkable advances have been achieved toward unravelling the complex hereditary etiopathogenesis underpinning AF, it has become increasingly clear that inherited determinants predisposing to AF in a vast majority of individuals are still uncertain. **Methods:** A Chinese pedigree with idiopathic AF and another group of 236 cases suffering idiopathic AF along with 312 unrelated healthy volunteers were prospectively recruited. Exome-wide sequencing and Sanger sequencing assays were implemented in research participants. The functional effects of the discovered variations in the *SOX5* gene were explored through dual-luciferase reporter analysis. **Results:** Two novel *SOX5* mutants, NM_006940.6: c.355C>T; p.(Gln119*) and NM_006940.6: c.640G>T; p.(Glu214*), were identified in the AF pedigree and one of the 236 unrelated patients affected with AF, respectively. These two heterozygous truncating SOX5 variations were absent from the 624 control chromosomes. Quantitative luciferase reporter assays unraveled that both Gln119*- and Glu214*-mutant SOX5 lost the ability to transactivate *GJA1*. Additionally, the two variations abolished the synergistic transactivation of *SCN5A* by SOX5 and SHOX2. **Conclusions:** The current findings indicate *SOX5* as a novel gene contributing to AF, which adds more insight to the molecular pathogenesis of AF, and provides a potential target for personalized precision medicine.

## 1. Introduction

As the third most prevalent cardiovascular disorder next to hypertension and coronary artery disease [1], atrial fibrillation (AF), characterized by irregular atrial electrical activity resulting in disorganized atrial contractions, is the most frequently sustained cardiac dysrhythmia, affecting about 1–2% of the general population worldwide [2,3]. According to a systematic scientific assessment of the global burden of diseases in 2019, over 43 million people suffered from AF globally [4], and in the United States alone, AF was estimated to affect more than five million people in 2010, with a projection of roughly 12 million in 2030 [2]. It has been reported that the total lifetime risk for the development of AF is ~30–40% in White folks, ~20% in African Americans, and ~15% in Chinese subjects [2]. Notably, nearly one-third of patients suffering from AF are asymptomatic (so-called silent/subclinical AF) and remain undetected until the first thromboembolic complication occurs; hence, the actual incidence of AF is evidently underestimated, given that intermittent screening with a conventional electrocardiogram may miss some episodes of AF because of its paroxysmal nature [5,6]. Although the factual prevalence of undiagnosed AF is unclear in the community, a retrospective cohort investigation has revealed that about 11% of the AF patients were undiagnosed in the United States in 2015 [7]. Recently, an assessment of asymptomatic AF in the general population shows that the true prevalence of AF is at a minimum of 3–4% if asymptomatic/device-detected AF is encompassed [8].

By disruption of atrial coordinated contraction [9], AF confers strikingly enhanced risks for a wide range of adverse clinical sequelae, encompassing reduced exercise capacity along with degraded health-correlated quality of life [10,11,12,13,14], atrial thrombosis [15,16,17,18] and thromboembolic cerebral stroke/systemic thromboembolism [19,20,21,22,23,24], cognitive impairment and early-onset dementia [25,26,27], acute renal injury/chronic kidney disease [28,29,30], atrial cardiomyopathy [31,32,33,34], myocardial infarction [35,36,37,38], congestive/chronic heart failure [39,40,41,42], malicious/lethal ventricular arrhythmias [43,44,45], and even premature cardiac demise [46,47,48,49]. More specifically, AF is associated with a 1.5-time risk of acute myocardial infarction or intellectual decline/dementia, 1.6-time risk of chronic renal disorder, 2.4-time risk of cerebral stroke, 5-time risk of congestive cardiac failure, and 2-time risk of sudden cardiac demise [2]. According to a retrospective investigation of a nationally representative population with Medicare beneficiaries, after the initial diagnosis of AF in persons ≥ 65 years, during the first five years, the most frequent clinical consequence was death, followed by cardiac failure, stroke, gastrointestinal hemorrhage, and acute myocardial infarction [50]. Additionally, AF is responsible for higher healthcare expenditures [2]. Based on the US data from Optum, subjects with incident AF have annual healthcare disbursements of $63,031, which is $27,896 higher than those without AF [51]. Evidently, AF has given rise to substantial morbidity and mortality as well as huge economic encumbrance [2,52,53,54]. Despite the vital clinical significance, the etiopathogenesis underlying AF remains largely indefinite.

It is generally understood that the etiopathogenesis underpinning AF is exceedingly complicated and multifaceted, and both environmental/non-hereditary pathogenic factors and genetic/inheritable defective components may lead to the occurrence and maintenance of AF [2,55,56,57,58,59]. There is a growing body of non-genetic hazard factors reported to account for AF, such as advancing age, unhealthy/sedentary lifestyle, night shift work, immoderate smoking, excessive alcohol consumption, obstructive sleep apnea, obesity, surgical operations, clonal haematopoiesis, diabetes mellitus, epilepsy, depression, gut microbiota dysbiosis, homocysteine, hyperuricaemia, asthma, pulmonary embolism, hyperthyroidism, bacterial/viral infections, β-thalassemia, chronic kidney disease, essential hypertension, rheumatic/valvular heart disease, coronary heart disease, dilated cardiomyopathy, hypertrophic cardiomyopathy, acoustic/toxicant pollution, and cardiac autonomic nerve system disorder [2,55,56,60,61,62,63,64,65]. However, aggregating genetic investigations have demonstrated that heritable determinants exert a crucial effect on the occurrence and sustainment of AF [57,58,59]. At present, no less than 60 AF-causing genes have been reported, of which the overwhelming majority encode sodium ion channel proteins, such as SCN5A (Na^+^ channel alpha subunit 5) and SCN1B (Na^+^ channel beta subunit 1); potassium ion channel subunits, such as KCNQ1 (K^+^ channel subfamily Q member 1) and KCNE2 (K^+^ channel subfamily E regulatory subunit 2); calcium ion channel proteins, such as CACNB2 (Ca^2+^ channel auxiliary subunit beta 2) and CACNA2D4 (Ca^2+^ channel auxiliary subunit alpha2delta 4); gap junction channel proteins/connexins, such as GJA1/Cx43 (gap junction protein alpha 1/connexin 43) and GJA5/Cx40 (gap junction protein alpha 5/connexin 40); transcription factors, such as PITX2 (paired like homeodomain 2) and SHOX2 (short stature homeobox 2); myocardial structural proteins, such as myosin light chain 4 (MYL4) and titin (TTN); and signaling molecules such as atrial natriuretic peptide (ANP) [57,58,59,66,67,68,69,70,71,72,73,74]. In addition, extensive genome- and exome-wide comparison assays between AF patients and controls, such as whole-exome sequencing (WES) analysis, have unveiled genetic variants at >140 chromosomal loci that confer enhanced susceptibility to AF, though the functional effects of these AF-related genetic variants remain largely unclear [59,75]. Nevertheless, due to noteworthy genetic heterogeneity, the hereditary ingredients susceptible to AF in most patients are still obscure.

## 2. Materials and Methods

### 2.1. Research Individuals

In the current research program, a four-generation pedigree suffering from idiopathic AF (arbitrarily named as Pedigree AF-001) and another group of 236 cases who were unrelated and suffered idiopathic AF, along with 312 healthy volunteers who were unrelated and had no familial history of AF, were prospectively enlisted from the population of the Han race in Shanghai, China. The criteria for the inclusion of AF patients were a definite diagnosis of AF, no known cause of AF, and informed consent. The criteria for the exclusion of AF patients included a definite environmental/genetic cause of AF and no informed consent. Diagnostic ascertainment and clinical classification of AF were conducted as elaborated previously [2,76]. Specifically, the diagnostic criteria for AF were based on the ESC 2020 guidelines, and idiopathic AF was defined without structural heart disease or systemic disorder [2,76]. Phenotypic data, which included personal information on medical history as well as familial history, medical records, physical examination findings, 12-lead electrocardiograms, trans-thoracic echocardiographic parameters, and routine laboratory test reports, were collected from each study subject. A 24-h ambulatory electrocardiographic monitoring was carried out when indicated. The control individuals underwent electrocardiographic and echocardiographic screening to confirm the absence of AF and were age- and sex-matched to AF patients. Blood samples were prepared from research participants, which were used to extract genomic DNA utilizing a DNA purification kit (catalogue No. A1620; Promega, Madison, WI, USA), following standard protocols. The current case-control investigation was conducted strictly in accordance with the Declaration of Helsinki. Before the enrollment of research participants, the Medical Ethics Committee at Shanghai Fifth People′s Hospital approved the study protocols (ethical approval code: 2022-179; ethical approval date: 23 October 2024). All research participants were of Chinese origin and signed a written informed consent form before the start of clinical investigations.

### 2.2. Genetic Assay in Study Participants

As described elsewhere [68,73,77,78,79], WES analysis was performed in five AF-affected members and three unaffected/healthy members from Pedigree AF-001. Briefly, 5 µg of genomic DNA from each family member selected for WES analysis were applied to construct a genomic DNA library, from which an exome library was constructed via hybridization capture utilizing the SureSelectXT2 Human All Exon (V6) Kit (catalogue No. 5190-8872; Agilent Technologies, Santa Clara, CA, USA). Sequencing of an exome library was implemented using the HiSeq 4000 Genome Analyzer (Illumina, San Diego, CA, USA), with the HiSeq 3000/4000 SBS Kit (catalogue No. 410-1003; Illumina, USA), according to the manufacturer’s protocols. Raw WES data were treated using Illumina Analysis Pipeline (version 2.6; Illumina, USA) with default settings, and reads were mapped to the human reference genome (GRCh37) with BWA (version 2.10.12). Variant calling was completed by leveraging GATK (version 3.8.1.0), and variant annotation was made by utilizing ANNOVAR (version 2018). Rare deleterious variations linked to AF were subject to Sanger sequencing assays in all the family members from Pedigree AF-001. Rare deleterious variations were based on such criteria as: with a minor allele frequency (MAF) < 0.01 in gnomAD/dbSNP, and predicted pathogenicity using in silico tools including SIFT, PolyPhen-2, and CADD. Sanger sequencing examination of the entire coding regions, as well as splicing donors/acceptors, of the gene harboring the observed AF-linked variation was implemented in another group of 236 patients with idiopathic AF, along with 312 unrelated healthy volunteers as controls. Besides, the dbSNP (accessed on 1 September 2025) and gnomAD (version 2.1.1; accessed on 1 September 2025) databases were consulted as described elsewhere [73] to verify the novelty of the discovered AF-linked variation.

### 2.3. Recombination of Gene-Expressing Vectors

Human heart cDNA was prepared as depicted previously [80,81]. A 2400-bp DNA segment harboring the whole open read frame of the wild-type human sex-determining region (SRY)-related high-mobility-group (HMG) box 5 (SO*X5*) gene (Nucleotide accession No.: NM_006940.6) was amplified through polymerase chain reaction (PCR) from human heart cDNA with the AccuPrime™ *Pfx* DNA Polymerase Kit (catalogue No. 12344024; Invitrogen, Carlsbad, CA, USA) using the *SOX5*-specific primers of 5′-CGCGAATTCACTTGACAGGTTCAGTTGGAG-3′ and 5′-CGCCTCGAGTCTTTAAGTCCTAAGGTCAC-3′. The PCR-yielded products and the pcDNA™3.1^(+)^ vector (catalogue No. V79020; Invitrogen, USA) were cut with *EcoR*I (catalogue No. R0101V; New England Biolabs, Ipswich, MA, USA) and *Xho*I (catalogue No. R0146V; New England Biolabs, USA), fragmented via 1.6% agarose gel electrophoresis, extracted utilizing the MinElute Gel Extraction Kit (catalogue No. 28604; Qiagen, Hilden, Germany) and ligated with the T_4_ DNA ligase (catalogue No. M0202V; New England Biolabs, USA) to produce the wild-type human SOX5-pcDNA™3.1^(+)^ recombinant vector. Using the wild-type human SOX5-pcDNA™3.1^(+)^ vector as a PCR template, the Gln119*-mutant SOX5-pcDNA™3.1^(+)^ vector was created via site-targeted mutagenesis employing a site-directed mutagenesis kit (catalogue No. 210518; Agilent Technologies, USA) and the forward primer of 5′-GAAGAAGGTGGGCGATAGAGTGGCGAGTCCTTG-3′ as well as the reverse primer of 5′-CAAGGACTCGCCACTCTATCGCCCACCTTCTTC-3′. Similarly, the Glu214*-mutant human SOX5-pcDNA™3.1^(+)^ vector was produced by site-targeted mutagenesis with the primers comprising 5′-CTGACCAGCCTCCGATAGCAGCTGTTGGCTGCC-3′ (forward) and 5′-GGCAGCCAACAGCTGCTATCGGAGGCTGGTCAG-3′ (backward). The SHOX2-pcDNA™3.1^(+)^ vector expressing wild-type human SHOX2 was constructed as described elsewhere [82]. The SOX9-pcDNA™3.1^(+)^ vector expressing wild-type human SOX9 (Nucleotide accession No.: NM_000346.4) was constructed like constructing the SOX5-pcDNA™3.1^(+)^ vector, using the primers of 5′-GTAGCTAGCGAAAGCGGAGCTCGAAACTG-3′ and 5′-GTAGCGGCCGCCAAGTGGGTAATGCGCTTGG-3′. Additionally, the GJA1-luciferase (GJA1-luc) and SCN5A-luc vectors expressing Firefly luciferase were constructed as described elsewhere [73]. All the recombinant vectors were verified by Sanger sequencing analysis.

### 2.4. Cellular Transfection with Multiple Expression Vectors and Dual-Luciferase Activity Measurement

COS7 and HEK293 cells were cultured as previously narrated [73]. Cells were transiently transfected with multiple expressing vectors utilizing a lipofectamine reagent (catalogue No. 15338100; Invitrogen, USA). Specifically, COS-7 cells were transiently transfected with 400 ng of empty pcDNA™3.1^(+)^ as an external control, or 400 ng of wild-type human SOX5-pcDNA™3.1^(+)^, or 400 ng of Gln119*-mutant human SOX5-pcDNA™3.1^(+)^, or 400 ng of Glu214*-mutant human SOX5-pcDNA™3.1^(+)^, or 200 ng of wild-type human SOX5-pcDNA™3.1^(+)^ + 200 ng of empty pcDNA™3.1^(+)^, or 200 ng of wild-type human SOX5-pcDNA™3.1^(+)^ + 200 ng of Gln119*-mutant human SOX5-pcDNA™3.1^(+)^, or 200 ng of wild-type human SOX5-pcDNA™3.1^(+)^ + 200 ng of Glu214*-mutant human SOX5-pcDNA™3.1^(+)^, together with 1.2 μg of GJA1-luc and 3 ng of pGL4.75 expressing Renilla luciferase (catalogue No. E6931; Promega, Madison, WI, USA). To analyze the synergistic transactivation function, HEK293 cells were transiently transfected with 200 ng of each expression vector (empty pcDNA™3.1^(+)^, wild-type human SOX5-pcDNA™3.1^(+)^, wild-type human SHOX2-pcDNA™3.1^(+)^, Gln119*-mutant human SOX5-pcDNA™3.1^(+)^, or Glu214*-mutant human SOX5-pcDNA™3.1^(+)^) together with 1.0 μg of SCN5A-luc and 2 ng of pGL4.75 (catalogue No. E6931; Promega, USA). Notably, in each cellular transfection, the SOX9-pcDNA™3.1^(+)^ vector (200 ng) was used together, due to its fundamental role for SOX5 function [83,84,85,86]. Cells were collected 48 h after transfection with the above-mentioned expression vectors, and then the activities of dual luciferases were quantitatively assayed using a dual-luciferase assay kit (catalogue No. E2920; Promega, USA) under a fluorescent plate reader (Promega, USA), following the manufacturer’s instructions. As depicted elsewhere [73], the activities of the *GJA1* and *SCN5A* promoters were presented as the ratios of Firefly bioluminescence intensities to Renilla bioluminescence intensities. In addition, for every vector used, three independent dual-reporter assays were performed in triplicate, and the means of results from three independent dual-reporter assays were applied to the comparison between two or among no less than three groups.

### 2.5. Statistical Assessment

Continuous parameters, such as age and promoter activity, are described as mean ± standard deviation ( $\bar{x}$ ± SD), whereas categorical parameters, such as sex and familial history of AF, are presented as counts/numbers as well as percentages. An unpaired Student’s *t*-test and a one-way analysis of variance followed by the Tukey–Kramer post-hoc test were utilized for continuous data to evaluate the differences between the two groups and among ≥3 groups, respectively. A Chi-square/χ^2^ or Fisher’s exact test was utilized for categorical data, as appropriate, to assess the differences between the two groups. A two-tailed *p* < 0.05 indicated a significant difference. Statistical analyses were conducted with the aid of SPSS (version 25.0; IBM, Armonk, NY, USA).

## 3. Results

### 3.1. Clinical and Demographic Characteristic Profiles of the Pedigree Members and Other Study Subjects

**Figure 1:**
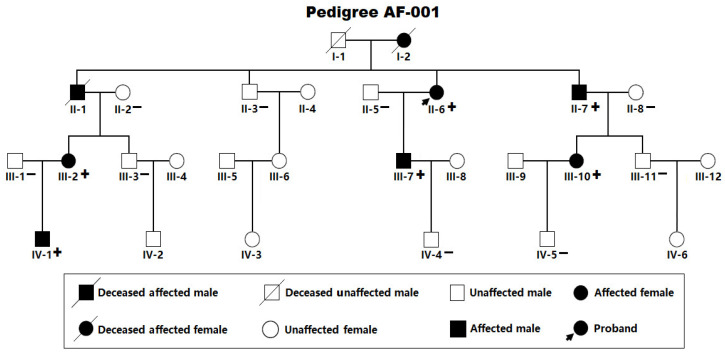
Pedigree with idiopathic atrial fibrillation. The family was named arbitrarily as Pedigree AF-001. “+” denotes an individual carrying the observed *SOX5* variation; “−” signifies a member without the found *SOX5* variation.

As portrayed in Figure 1, a four-generation pedigree comprising 28 members with idiopathic AF (arbitrarily termed Pedigree AF-001) was recruited, including 25 living pedigree members.

**Figure 2:**
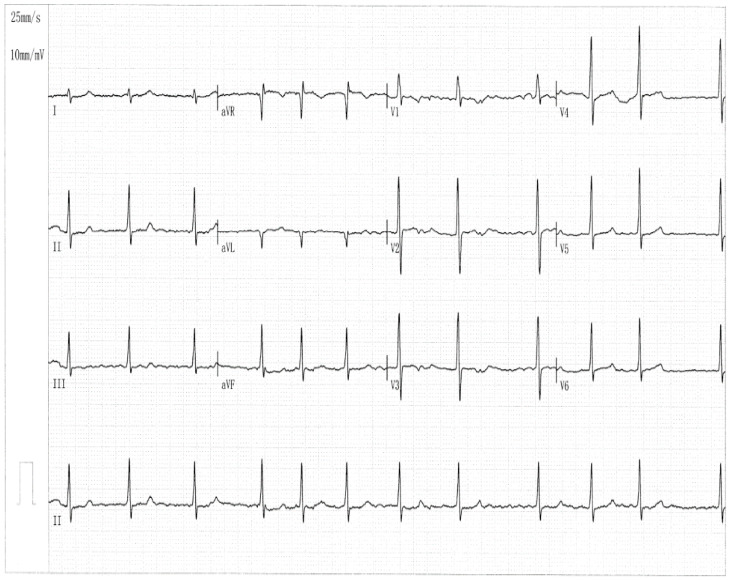
A representative 12-lead electrocardiogram from the proband (member II-6 in Pedigree AF-001). The standard electrocardiogram documents the occurrence of atrial fibrillation.

In Pedigree AF-001, eight members, encompassing four female and four male members, had a definite diagnosis of AF in terms of the electrocardiographic findings/medical records. No environmental/acquired risk factors prone to AF were detected in the members from Pedigree AF-001, such as obesity, obstructive sleep apnea, hyperthyroidism, coronary artery disease, essential arterial hypertension, dilated/hypertrophic cardiomyopathy, acute myocarditis, chronic heart failure, cardiac surgery, pulmonary heart disease, chronic kidney disease, and diabetes mellitus. The index patient (member II-6 from Pedigree AF-001), a 66-year-old female individual with 15 years of AF history, was referred to the local hospital due to an acute attack of syncope. One representative electrocardiogram showing AF of the index patient was provided in Figure 2.

The proband (member II-6 in Pedigree AF-001) underwent a successful catheter-based radiofrequency ablation for AF during this hospitalization. Her mother (member I-2 from Pedigree AF-001) had 24 years of AF history and died of an acute attack of cerebral stroke at 69 years of age. The index patient’s elder brother (member II-1 from Pedigree AF-001) had 20 years of AF history and died of stroke at the age of 64 years. The proband’s younger brother (member II-7 in Pedigree AF-001), a 63-year-old male member with 11 years of AF history, underwent a successful radiofrequency ablation of AF when he was 58 years old. The index case’s elder niece (member III-2 from Pedigree AF-001), a 48-year-old member with four years of AF history, underwent a successful radiofrequency ablation of AF at the age of 46 years. The index case’s other relatives affected with AF had a medical history of taking antiarrhythmic drugs, but did not undergo surgical/catheter-based therapy for AF until recruitment. The index case’s unaffected relatives, including 11 male and nine female individuals, had no history of AF episode, with normal electrocardiograms. The basic clinical and demographic characteristic profiles of the pedigree members suffering AF are narrated in Table 1.

Additionally, all the pedigree members suffering AF also manifested intellectual impairment, developmental delay of language, and diverse facial dysmorphisms, including broad nasal bridges, wide mouths, and tooth anomalies. The index case’s elder brother (member II-1 from Pedigree AF-001) had also episodes of seizures.

Additionally, another group of 236 cases affected with idiopathic AF underwent clinical investigation, in contrast to 312 healthy persons with no familial history of AF, who were enlisted as control subjects. The demographic and baseline clinical characteristic data of this cohort of AF cases, along with the control people, are described in Table 2.

### 3.2. Identification of Two Novel SOX5 Variations Contributing to AF

**Figure 3:**
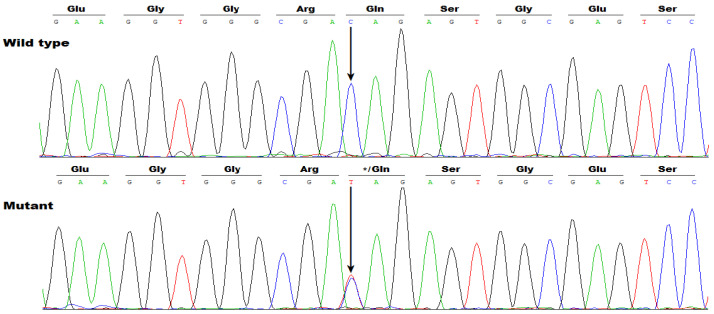
A novel *SOX5* variation accountable for atrial fibrillation. Sequencing electropherograms delineating the heterogeneous SOX5 variation discovered in the proband with atrial fibrillation (Mutant), along with its wild-type control from a healthy person (Wild type). An arrow pinpoints where the variation occurs. * means a stop codon.

WES analysis was completed in six AF members (II-6, II-7, III-2, III-7, III-10 and IV-1) and seven healthy members (II-2, II-3, II-5, II-8, III-1, IV-4 and IV-5) from Pedigree AF-001 (Figure 1), by which only the pathogenic variation of chr12: 23,999,043C>T (GRCh37.p13/GCF_000001405.25/hg19: NC_000012.11), equal to chr12: 24,246,045C>T (GRCh38.p14/GCF_000001405.40/hg38: NC_000012.12) or NM_006940.6: c.355C>T; p.(Gln119*), was discovered to co-segregate with AF, and verified by Sanger sequencing assays to be in co-segregation with AF in the entire family (Pedigree AF-001). The sequencing chromatogram traces illustrating the heterozygous c.355C>T variation in *SOX5*, along with its wild type as a sequence control, are provided in Figure 3.

Moreover, Sanger sequencing examination of the entire coding regions and splicing donors/acceptors of *SOX5* was implemented in all the research participants utilizing the primer pairs presented in Table 3, which confirmed that the variation of NM_006940.6: c.355C>T; p.(Gln119*) in *SOX5* was shared by all the AF family members but by none of the unaffected family members of Pedigree AF-001. Genetic analysis of Pedigree AF-001 indicated that AF was transmitted in an autosomal-dominant mode.

**Figure 4:**
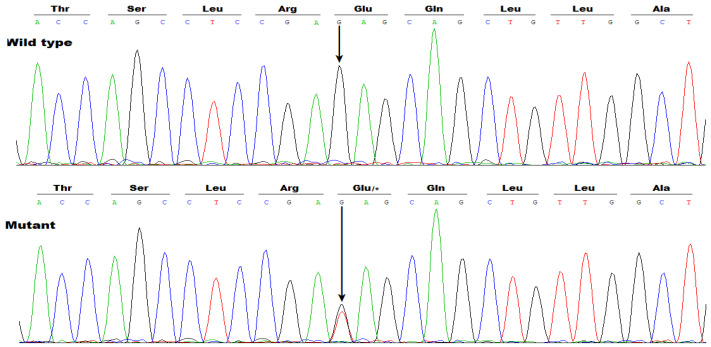
A de novo *SOX5* variation predisposing to atrial fibrillation. The heterogeneous *SOX5* variation (Mutant) was detected in one of 236 patients with atrial fibrillation, and its wild type as a sequence control was detected in a healthy subject (Wild type). A vertical arrow orients the nucleotide site where the variation occurs. * means a stop codon.

Additionally, Sanger sequencing examination of the whole coding regions along with splicing junction sites of the *SOX5* gene in another group of 236 cases suffering from idiopathic AF unveiled a heterozygous *SOX5* variation of NM_006940.6: c.640G>T; p.(Glu214*), residing in the fifth coding exon of *SOX5* and resulting in a conversion of glutamic acid codon to stop codon at amino acid position 214 of SOX5, in one male case who was aged 41 years, had no family history of AF, but also suffered intellectual impairment, delayed language development, and mild facial dysmorphisms characterized by broad nasal bridges and teeth abnormalities. This *SOX5* variation was not observed in his parents, who had normal electrocardiograms without AF, indicating a de novo mutation. The sequencing chromatogram traces exhibiting the c.640G>T variation in *SOX5*, together with its wild type as a sequence control, are given in Figure 4.

**Figure 5:**
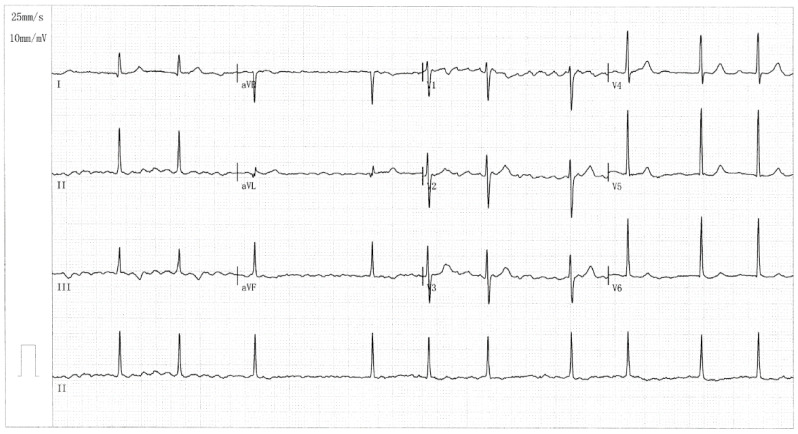
A representative 12-lead electrocardiogram recorded from the AF case carrying the *SOX5* c.640G>T variation. This electrocardiogram manifests atrial fibrillation.

A representative electrocardiogram indicating AF from the case harboring the *SOX5* c.640G>T variation is shown in Figure 5.

Neither of the two identified *SOX5* mutations responsible for AF was found in the 624 human control chromosomes, or in the databases of gnomAD and dbSNP, confirming the novelty of the two *SOX5* mutations.

### 3.3. Functional Failure of Gln119*- or Glu214*-Mutant SOX5 to Transactivate GJA1

**Figure 6:**
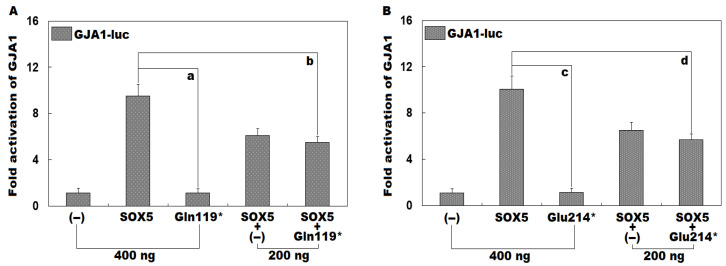
Failure of Gln119*- or Glu214*-mutant SOX5 to transactivate *GJA1*. In cultured COS-7 cells in vitro, dual-reporter (Firefly luciferase and Renilla luciferase) gene analysis revealed that both the Gln119* mutant (**A**) and the Glu214* mutant (**B**) failed to transcriptionally activate *GJA1*. Herein, “a” and “c” signify *p* < 0.001, and “b” and “d” denote *p* < 0.005, in comparison with wild-type human SOX5 (400 ng). + means plus, − means empty plasmid (negative control).

As displayed in Figure 6A, in cultivated COS-7 cells transfected with multiple expression plasmids, encompassing empty pcDNA™3.1^(+)^ plasmid (−), wild-type human SOX5-pcDNA™3.1^(+)^ plasmid (SOX5), and Gln119*-mutant human SOX5-pcDNA™3.1^(+)^ plasmid (Gln119*), singly or together, SOX5 and Gln119* induced transactivation of *GJA1* by ~9-fold and ~1-fold, respectively (SOX5 vs. Gln119*: t = 13.3255; *p* = 0.0002). When SOX5 and Gln119* were transfected together, the induced transcriptional activation of *GJA1* was ~6-fold (SOX5 vs. Gln119* + SOX5: t = 6.0364; *p* = 0.0038). Additionally, similar results were given when multiple comparisons were performed (F = 96.4652, *p* = 6.045 × 10^−8^). Specifically, for (−) vs. SOX5, t = 8.3967; *p* < 0.0001; for (−) vs. Gln119*, t = 0.0367; *p* = 1.0000; for (−) vs. SOX5 + (−), t = 4.9753; *p* < 0.0001; for (−) vs. SOX5 + Gln119*, t = 4.4033; *p* < 0.0001; for SOX5 vs. Gln119*, t = 8.3600; *p* < 0.0001; for SOX5 vs. SOX5 + (−), t = 3.4213; *p* = 0.0004; for SOX5 vs. SOX5 + Gln119*, t = 3.9933; *p* = 0.0001; for Gln119* vs. SOX5 + (−), t = 4.9387; *p* < 0.0001; for Gln119* vs. SOX5 + Gln119*, t = 4.3667; *p* < 0.0001; for SOX5 + (−) vs. SOX5 + Gln119*, t = 0.5720; *p* = 0.07978. Similarly, as shown in Figure 6B, SOX5 and Glu214* transcriptionally activated *GJA1* by ~10-fold and ~1-fold, respectively (SOX5 vs. Glu214*: t = 12.9871; *p* = 0.0002). When SOX5 and Glu214* were transfected in combination, the induced transactivation of *GJA1* was ~6-fold (SOX5 vs. Glu214* + SOX5: t = 6.0837; *p* = 0.0037). Besides, equal statistical results were generated when multiple comparisons were conducted (F = 97.2192, *p* = 5.818 × 10^−8^). Specifically, for (−) vs. SOX5, t = 8.9833; *p* < 0.0001; for (−) vs. Glu214*, t = 0.0733; *p* = 0.9999; for (−) vs. SOX5 + (−), t = 5.4533; *p* < 0.0001; for (−) vs. SOX5 + Glu214*, t = 4.6233; *p* < 0.0001; for SOX5 vs. Glu214*, t = 8.91; *p* < 0.0001; for SOX5 vs. SOX5 + (−), t = 3.53; *p* = 0.0006; for SOX5 vs. SOX5 + Glu214*, t = 4.36; *p* < 0.0001; for Glu214* vs. SOX5 + (−), t = 5.38; *p* < 0.0001; for Glu214* vs. SOX5 + Glu214*, t = 4.55; *p* < 0.0001; for SOX5 + (−) vs. SOX5 + Glu214*, t = 0.83; *p* = 0.5779.

### 3.4. Inability of Gln119*- or Glu214*-Mutant SOX5 to Induce Transactivation of SCN5A Singly or Synergistically with SHOX2

**Figure 7:**
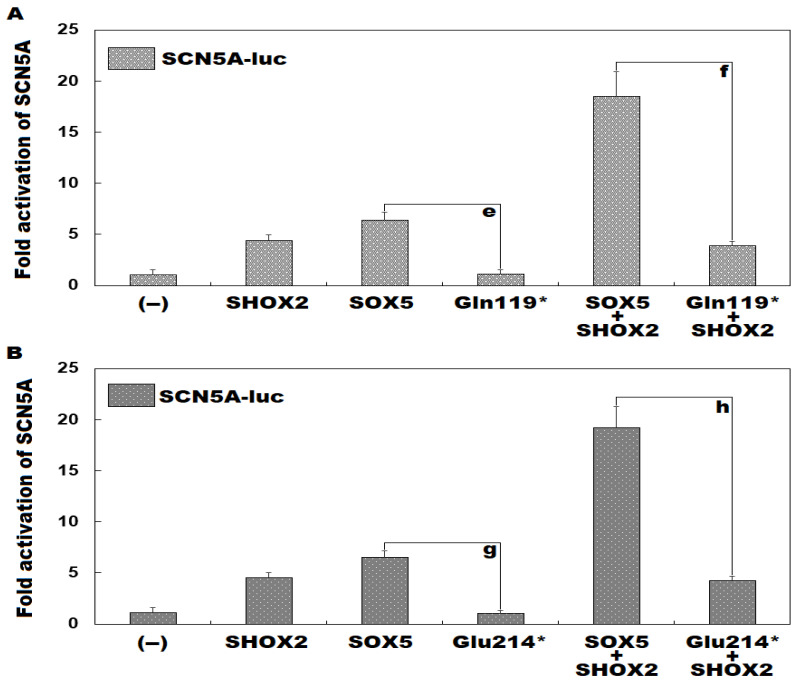
Synergistic activation of *SCN5A* between SOX5 and SHOX2 disrupted by the Gln119*- or Glu214* mutation. In the HEK293 cells maintained in vitro and transfected with multiple expression plasmids, dual-luciferase activity measurement demonstrated that the synergistic activation of *SCN5A* by SOX5 and SHOX2 was disrupted by the Gln119* mutation (**A**) or the Glu214* mutation (**B**). Herein, “e”, “f”, “g”, and “h” all mean *p* < 0.001, in contrast to the corresponding wild-type counterparts. + means plus, − means empty plasmid (negative control).

As exhibited in Figure 7A, in HEK-293 cells cultivated in vitro expressing multiple plasmids, including empty pcDNA™3.1^(+)^ plasmid (−), wild-type human SHOX2-pcDNA™3.1^(+)^ plasmid (SHOX2), wild-type human SOX5-pcDNA™3.1^(+)^ plasmid (SOX5), and Gln119*-mutant human SOX5-pcDNA™3.1^(+)^ plasmid (Gln119*), separately or in both, SOX5 and Gln119* induced transactivation of *SCN5A* by ~6-fold and ~1-fold, respectively (SOX5 vs. Gln119*: t = 10.0999; *p* = 0.0005). Together with SHOX2, SOX5 and Gln119* transcriptionally activated *SCN5A* by ~18-fold and ~4-fold, respectively (SOX5 + SHOX2 vs. Gln119* + SHOX2: t = 10.1226; *p* = 0.0005). In addition, similar results were obtained if multiple comparisons were made (F = 100.0152, *p* = 2.356 × 10^−9^). Specifically, for (−) vs. SHOX2, t = 3.3533; *p* = 0.0310; for (−) vs. SOX5, t = 5.36; *p* = 0.0009; for (−) vs. Gln119*, t = 0.0633; *p* = 1.0000; for (−) vs. SOX5 + SHOX2, t = 17.45; *p* < 0.0001; for (−) vs. Gln119* + SHOX2, t = 2.8233; *p* = 0.0811; for SHOX2 vs. SOX5, t = 2.0067; *p* = 0.3131; for SHOX2 vs. Gln119*, t = 3.29; *p* = 0.0348; for SHOX2 vs. SOX5 + SHOX2, t = 14.0967; *p* < 0.0001; for SHOX2 vs. Gln119 + SHOX2, t = 0.53; *p* = 0.9909; for SOX5 vs. Gln119*, t = 5.2967; *p* = 0.0010; for SOX5 vs. SOX5 + SHOX2, t = 12.09; *p* < 0.0001; for SOX5 vs. Gln119 + SHOX2, t = 2.5367; *p* = 0.1339; for Gln119* vs. SOX5 + SHOX2, t = 17.3867; *p* < 0.0001; for Gln119* vs. Gln119 + SHOX2, t = 2.76; *p* = 0.0907; for SOX5 + SHOX2 vs. Gln119* + SHOX2, t = 14.6267; *p* < 0.0001. Similarly, as depicted in Figure 7B, SOX5 and Glu214* transcriptionally activated *SCN5A* by ~7-fold and ~1-fold, respectively (SOX5 vs. Glu214*: t = 14.1459; *p* = 0.0001). In the presence of SHOX2, SOX5 and Glu214* transcriptionally activated *SCN5A* by ~19-fold and ~4-fold, respectively (SOX5 + SHOX2 vs. Glu214* + SHOX2: t = 12.3580; *p* = 0.0002). In addition, similar results were generated if multiple comparisons were carried out (F = 153.8289, *p* = 1.889 × 10^−10^). In detail, for (−) vs. SHOX2, t = 3.4833; *p* = 0.0071; for (−) vs. SOX5, t = 5.46; *p* = 0.0001; for (−) vs. Glu214*, t = 0.007; *p* = 1.0000; for (−) vs. SOX5 + SHOX2, t = 18.1133; *p* < 0.0001; for (−) vs. Glu214* + SHOX2, t = 3.13; *p* = 0.0154; for SHOX2 vs. SOX5, t = 1.9767; *p* = 0.1804; for SHOX2 vs. Glu214*, t = 3.5533; *p* = 0.0061; for SHOX2 vs. SOX5 + SHOX2, t = 14.63; *p* < 0.0001; for SHOX2 vs. Glu214* + SHOX2, t = 0.3533; *p* = 0.9968; for SOX5 vs. Glu214*, t = 5.53; *p* = 0.0001; for SOX5 vs. SOX5 + SHOX2, t = 12.6533; *p* < 0.0001; for SOX5 vs. Glu214* + SHOX2, t = 2.33; *p* = 0.0874; for Glu214* vs. SOX5 + SHOX2, t = 18.1833; *p* < 0.0001; for Glu214* vs. Glu214* + SHOX2, t = 3.2; *p* = 0.0132; for SOX5 + SHOX2 vs. Glu214* + SHOX2, t = 14.9833; *p* < 0.0001.

## 4. Discussion

The SOX family of proteins comprises a highly conserved cluster of transcription factors characterized by harboring the HMG domain composed of three α-helices, which binds the core DNA sequence 5′-AACAAT-3′ in the promoters of downstream genes, regulating the expression levels of target genes [86]. This HMG domain of a SOX protein not only binds target DNA, but also mediates subcellular trafficking and interactions with transcriptionally cooperative partners/co-factors [86]. To date, in vertebrates, a group of 20 SOX proteins has been found, which are classified into eight subgroups (from SOXA to SOXH) in terms of the amino acid sequence conservation/identity within the HMG motif as well as the existence of other domains [86]. It has been demonstrated that SOX proteins exert a pivotal effect on the embryonic development of most organs and postnatal pathological processes in various tissues derived from the endoderm, mesoderm, and ectoderm, encompassing the cardiovascular system, brain, bone, cartilage, lymphatic system, retina, pancreas, and hematopoietic system [87,88]. In addition, it has been reported that genetically defective SOX proteins contribute to many genetic diseases, so-called ‘SOXopathies’, affecting the cardiovascular system, urinary system, central nervous system, muscular system, reproductive system, auditory and ocular systems, as well as skeleton, skin, and hair [87,88]. The SOXD subgroup of transcription factors includes SOX13, SOX6, and SOX5, of which SOX5 consists of 763 amino acids and contains two coiled-coil motifs (amino acids 193–274 and amino acids 448–493) located at the N-terminus and a family-restricted HMG domain (amino acids 555–630) located at the C-terminus [86,89]. The coiled-coil domain functions to regulate *SOXD* protein dimerization (homo- and hetero-dimerization) and promote preferential binding to the adjoining HMG recognition sites by adding flexibility [86,89]. The *SOX5* gene is mapped on human chromosome 12p12.1, which produces multiple transcript isoforms by alternative transcription start site and precursor messenger RNA splicing, including the longest isoform (originally named as *L-SOX5*; Nucleotide accession No.: NM_006940.6) and the shortest isoform (also termed as *S-SOX5*; Nucleotide accession No.: NM_178010.4) [86]. The longest isoform of *SOX5* encodes a protein with 763 amino acids (encoded by exons 1–15), which is amply expressed in various tissues, encompassing heart and brain, playing a critical role in the development and remodeling of cardiovascular and cerebrovascular systems, predominantly participating in cell proliferation, cell cycle regulation, cellular migration and invasion, cell apoptosis, and inflammatory response [86,90,91,92,93], while the shortest isoform of *SOX5* encodes a 377-amino-acid protein (encoded mainly by exons 10–15), which is specifically and highly expressed in the testis, playing a crucial role in the morphogenesis and function of motor cilia in the testes/spermatozoa [94,95,96]. The full-length *L-SOX5* is usually referred to as *SOX5*, and this appellation is consistently adopted in the references, mainly because *L-SOX5* is functionally and structurally equal to *SOX13* and *SOX6*, and contains a glutamine-rich region and a leucine zipper, which allows dimerization with other SOX proteins, such as SOX9, to cooperatively activate target genes [96]. In the current research, two new *SOX5* mutations linked to AF were discovered, including c.355C>T (p.Gln119*) locating at exon 3 and c.640G>T (p.Glu214*) locating at exon 5, hence were anticipated to produce truncating L-SOX5 proteins without HMG domain along with coiled-coil domain and fail to bind target promoters to transactivate downstream genes but have no effect on S-SOX5. Biological assay demonstrated that both the Gln119*- mutant SOX5 and the Glu214*-mutant SOX5 lost the ability to transactivate the expression of *GJA1*, an AF-causative gene [97]. Furthermore, both the Gln119*- mutant SOX5 and the Glu214*-mutant SOX5 failed to transactivate the expression of *SCN5A*, alone or synergistically with SHOX2, and pathogenic variations in both *SCN5A* and *SHOX2* have been discovered to result in AF [82,98,99,100,101]. Mechanistically, the two truncating mutations identified in our work were predicted to produce truncated SOX5 proteins without the HMG and coiled-coil domains, which supported the loss of transactivation of *GJA1* and *SCN5A* by the truncated SOX5 proteins observed experimentally, because the structural domains of HMG and coiled-coil are functionally indispensable for SOX5, and without them, the truncated SOX5 proteins could not bind to target gene DNA, or mediate subcellular trafficking and interactions with transcriptionally cooperative partners, hence lost transactivation function. Therefore, genetically compromised *SOX5* predisposes to AF at least in part by lowering the expression levels of its target genes, such as *SCN5A* and *GJA1*.

In humans, the correlation of genetic variations near the *SOX5* gene to AF has been clinically investigated. Olesen and coworkers [102] enlisted 209 patients suffering from AF and 534 control subjects without AF, and a total of 8 SNPs were genotyped in study participants by utilizing TaqMan assays. As a result, three SNPs were discovered to be associated with AF, including rs11047543 near *SOX5*, rs2200733 closest to *PITX2*, and rs3807989 adjacent to *CAV1*. Even if correction was made for multiple testing, rs11047543 and rs2200733 were both still associated with AF [102]. Pfeufer and colleagues [103] performed a meta-analysis of whole-genome association investigations for the hereditary determinants of electrocardiographic PR intervals and their relation to AF in 28,517 European-descent individuals from seven community-based studies. As a result, nine loci were found to be significantly associated with PR intervals, of which five loci were also significantly associated with AF, including rs11047543 near *SOX5* (51 kb 5′ of *C12orf67*), rs3807989 at intron 2 of *CAV1*/*CAV2*, rs11708996 at intron 14 of *SCN5A*, rs251253 next to *NKX2-5* (3 kb 5′ of *C5orf41*), and rs6800541 at intron 14 of *SCN10A* [102]. Park and partners [104] genotyped 16 SNPs (including rs11047543, rs2106261, rs6800541, rs13376333, rs2200733, rs3825214, rs10465885, rs3807989, rs853445, rs7193343, rs17042171, rs251253, rs10033464, rs11708996, rs17570669, and rs6843082) in a total of 89 Korean patients with early-onset and drug-refractory AF who experienced catheter-based ablation for AF, and observed that three SNPs, including rs11047543 closest to *SOX5* (12p12), rs3825214 neighboring *TBX5* (12q24), and rs7193343 adjacent to *ZFHX3* (16q22), were associated with the enhanced risk for the recurrence of AF after catheter-based radiofrequency ablation therapy, and the risk-allele number of these three SNPs could independently predict the recurrence of AF. Vogel et al. [105] examined the relationship between eight SNPs (located within or near the genes *SOX5*, *KCNN3*, *CAV1*, *PITX2*, *KCNJ5*, *ZFHX3*, and *MYH7*) and the risks of AF occurrence and recurrence in 259 AF patients and 108 control persons and revealed that the variation of rs11047543 near to *SOX5* conferred a higher risk on the recurrence of AF after treatment with direct current cardioversion. In addition, Seifert et al. [106] explored the correlation between the four SNPs previously implicated with AF and PR interval (rs11047543, rs3807989, rs13376333, and rs2200733) and the electrocardiographic P-wave morphology in 176 cases affected with AF, and found that two SNPs, including rs11047543 next to *SOX5* and rs3807989 in the vicinity of *CAV1*/*CAV2*, were significantly associated with abnormal P-wave morphology, implying significant effect on atrial conduction properties. Collectively, these observational results suggest that common genetic variations near *SOX5* are associated with the emergence and recurrence of AF, although the biological pathway/pathogenic mechanisms by which the above-mentioned SNPs lead to the development of AF remain to be experimentally elucidated.

It has been validated that SOX proteins, including SOX5, are involved in the regulation of multiple signaling pathways, and in the canonic WNT pathway, SOX5 functions as a key player to compete with T-cell factors/lymphoid enhancer factors for binding to β-catenin, resulting in the repression of the WNT/β-catenin pathway and hence the reduced expression levels of the WNT/β-catenin target genes [96,107]. The WNT pathway plays a key role in embryogenesis, tissue homeostasis, and a wide variety of pathophysiological processes, including the activation of adaptive cardiac remodeling and the increase of cardiac fibrosis [108,109]. Atrial fibrosis has been substantiated to be a hallmark of atrial structural remodeling and electrophysiological dysfunction/heterogeneous conduction, creating a pivotal substrate in favor of the initiation and perpetuation of AF [109,110,111], and ablation of fibrotic atrial areas has been demonstrated to improve the therapeutic results of catheter ablation for AF [112]. Additionally, SOX5 can also promote fibrosis by up-regulating the expression levels of N-fibronectin, cadherin, and vimentin [113]. Therefore, *SOX5* haploinsufficiency may predispose to AF, probably by increasing the WNT/β-catenin activity, generating an important matrix in favor of the occurrence and maintenance of AF.

The critical roles of *SOX5* in cardiac organogenesis and structural remodeling have been shown in animals [93,114,115,116]. In adult Drosophila models, knockdown of *Sox102F*, a fruit fly ortholog of human *SOX5*, led to a significant decrease in resting heart rate, ventricular wall velocity and cardiac chamber volume, along with a significant increase in ventricular wall thickness with disrupted myofibril structure and WNT signaling transduction [114]. In mice, knockout of *Sox5* led to neonatal lethality, with respiratory distress attributable to anomalous development of lungs, and mild skeletal anomalies, and double knockout of *Sox5* and *Sox6* resulted in murine embryonic death, with more severe pulmonary and skeletal abnormalities [115,116]. Unfortunately, the early death of *Sox5*-null mouse models prevented analyzing the effect of *Sox5* on adult murine cardiac function [115,116]. In the murine hearts with doxorubicin-induced dilated cardiomyopathy, the expression of SOX5 was increased, the WNT/β-catenin pathway and apoptosis were activated, and inflammation and collagen deposition were also increased, which were consistent with the findings from the hearts of patients with dilated cardiomyopathy [93]. In addition, in the hearts, the action potential elicits Ca^2+^ entry into cardiac myocytes via L-type Ca^2+^ channels, while in murine myoblast cells, knockdown of *Sox5* led to a significant decrease in the maximum charge movement, generated by voltage-gated L-type Ca^++^ channels [117]. In endothelial cells, SOX5 regulates shear stress-regulated gene expression in a nitric oxide-dependent mode [118], and nitric oxide is a key molecule for endothelial and cardiovascular function, which has been associated with AF [119]. Collectively, these investigations indicate that SOX5 may regulate voltage-gated L-type Ca^2+^ channels, and that genetically defective SOX5 may contribute to AF through altering atrial action potential and WNT signaling transduction, as well as atrioventricular conduction.

Previously in humans, dozens of deleterious *SOX5* variations, encompassing nonsense, missense and frame-shifting variations, were implicated in the etiopathogenesis of Lamb–Shaffer Syndrome, an uncommon genetic disease with a wide spectrum of clinical manifestations, including intellectual impairment/disability, developmental delay of language, attention deficits, seizures, hypotonia, autism spectrum disorder, hyperactivity, scoliosis, visual problem/strabismus, short stature, abnormal hands/feet, and diverse facial dysmorphisms, such as a bulbous nasal tip, a wide mouth, frontal bossing, deep-set eyes, prominent philtra ridges, and epicanthal folds [86,95,120]. In the current research, two novel *SOX5* variations, NM_006940.6: c.355C>T; p.(Gln119*) and NM_006940.6: c.640G>T; p.(Glu214*), were causally linked to AF as a prominent clinical manifestation of Lamb–Shaffer syndrome, therefore expanding the *SOX5*-related phenotypic spectrum. Given that the larger part of AF occurs paroxysmally or sub-clinically with no apparent symptoms [2], the present investigation suggests that long-term dynamic electrocardiographic screening of the cases suffering from Lamb–Shaffer syndrome attributed to *SOX5* variations is needed for the timely diagnosis of AF.

There exist potential limitations to the present investigation. Firstly, the study participants included only individuals of the Chinese Han ethnicity, and to date, no other *SOX5* loss-of-function variants have been found in AF patients of different origins in such public databases as gnomAD; therefore, our results might not apply to different populations. Obviously, identification of *SOX5* variants in different populations will strengthen the causal links of mutant *SOX5* to AF. Secondly, by WES analysis, a clinical diagnosis was made only in approximately 30% of cases referred for presumed genetic diseases [121], which underscored the limitations of WES itself, encompassing limited bioinformatical tools, the presence of some genes with unclear biological significance, and the inability to analyze non-coding regions. Thirdly, there is a need for further experimental evidence that the functional effects of mutant *SOX5* are associated with AF pathogenesis. Finally, further genetic exploration of the other 235 cases from the study AF cohort is needed in the future, which may discover new AF-causing genes.

## 5. Conclusions

The current study identified two truncating mutations, confirmed loss of transcriptional activity, and thereby established a functional link between *SOX5* and AF. The data indicate *SOX5* as a new gene contributing to AF, which adds insight into the molecular mechanism underpinning AF, and offers a molecular target for genetic counseling and potential individualized medical management of AF in a subset of cases. Nevertheless, further studies are needed to validate the findings in larger and diverse cohorts or to investigate the biological effects of *SOX5* variants in vivo.

## Figures and Tables

**Table 1:** Demographic and baseline clinical characteristics of the living pedigree members affected with atrial fibrillation.

		Individual Information		Cardiac Manifestation	Electrocardiogram			Echocardiogram	
Identity (Pedigree AF-001)	Sex	Age at First Diagnosis of AF (Years)	Age at Enrollment (Years)	AF (Clinical Categorizing)	Heart Rate (Beats/min)	QRS Interval (ms)	QTc (ms)	LAD (mm)	LVEF (%)
II-6	F	51	66	LSP	69	86	435	39	58
II-7	M	47	63	LSP	76	118	507	36	62
III-2	F	42	48	LSP	105	92	482	38	60
III-7	M	35	42	LSP	83	95	394	33	63
III-10	F	40	40	Persistent	112	105	416	32	64
IV-1	M	24	24	Paroxysmal	90	81	413	29	66

F, female; M, male; AF, atrial fibrillation; QTc, corrected QT interval; LSP, long-standing persistent; LVEF, left ventricular ejection fraction; LAD, left atrial diameter.

**Table 2:** Baseline clinical and demographic characteristic data of the group of 236 patients suffering atrial fibrillation, along with the 312 control subjects.

Variable	Case Group (*n* = 236)	Control Group (*n* = 312)	*p*-Value
Sex (male/female)	135/101	178/134	0.9716
Age (years)	54.16 ± 7.39	53.82 ± 8.02	0.6115
Family history of atrial fibrillation (%)	48 (20.33)	0 (0)	<0.0001 *
History of cerebral stroke (%)	15 (6.36)	0 (0)	<0.0001 *
History of implanting a pacemaker (%)	12 (5.08)	0 (0)	<0.0001 *
Body mass index (kg/m^2^)	22.85 ± 3.10	23.06 ± 2.96	0.4207
Total cholesterol (mmol/L)	4.15 ± 0.57	4.21 ± 0.61	0.2415
Fasting blood glucose (mmol/L)	4.46 ± 0.62	4.50 ± 0.69	0.4832
Triglyceride (mmol/L)	1.38 ± 0.35	1.40 ± 0.31	0.4797
Systolic blood pressure (mmHg)	127.84 ± 9.07	128.18 ± 9.41	0.6707
Diastolic blood pressure (mmHg)	84.73 ± 7.46	85.03 ± 9.02	0.6785
Resting heart rate (beats/min)	77.01 ± 8.47	76.88 ± 7.64	0.8508
Left ventricular ejection fraction (%)	62.25 ± 7.13	62.91 ± 7.22	0.2872
Left atrial diameter (mm)	37.82 ± 6.52	36.05 ± 6.08	0.0011 *

* *p* < 0.05.

**Table 3:** Primers to amplify the whole coding exons along with splicing donors/acceptors of the human *SOX5* gene.

Coding Exon	Forward Primer (5′→3′)	Backward Primer (5′→3′)	Amplicon (bp)
1	GGTTGTCTAGAGCCTTGCAGC	TTTGGTCCGGGCAATCACAAC	541
2	GTTCTGTTGCTACCTGCTTGGC	CTAAGACGCCAGGGGTGAATC	555
3	TCAGCTGAATAAGCCATATAACC	CAAGCAGGTGACTATTCCCG	660
4	GAAGTGGGGCTGGGATAGGG	GAAGCAGAAGAGGTGAGGGCA	300
5	TTATTTCCAGCTGGCCCTAGCAT	TGTTGTGTGCCTAGGACAGTGA	656
6	CTGCCGTGGTATCTTAGGCTTC	TGGTTCCCTGCACCTATCCAG	638
7	TGGGAAGAAGCATGGAGCATC	ATGATGCGAGTCCAGAGTCAAGA	648
8	AAAAGGATGAGGTTTCCGCCT	TTGTTAAGTCGCCTTGCTCCT	678
9	TGTTTCGGGTGCCCATTTCAAG	AGCTGCTGGCATACAATAGACA	681
10	TTTGATGGGAAATGACAGGCTGC	AAACGGACCTAGGTGGTTCCTC	418
11	GGCCAGACACTACCTATTACCAAGA	ACAAGCTGGTGGCGTAAAAGG	500
12	GGCATACCAAACCCAAACGCC	AATGATGAGGTATGAGGTGGCTG	456
13	CATTTGCCACCACAAGGCTTATC	ATCCAGGATCCTTCCACAACTGC	440
14	AGGTACAAAACCACCACCACCT	TGGTAGAGCTAGGAACTTGCAGTG	592
15	ACATCTAACTATTCACTTACCCACG	GTGCTTGGCCACTGGTAAGG	435

## Data Availability

The original contributions presented in this study are included in the article. Further inquiries can be directed to the corresponding authors.
